# Co-occurrence of depressive symptoms, risk of malnutrition, and functional limitations in chronically ill patients receiving long-term home care

**DOI:** 10.3389/fpsyt.2026.1753827

**Published:** 2026-03-09

**Authors:** Bożena Majchrowicz, Krystyna Kowalczuk, Katarzyna Tomaszewska

**Affiliations:** 1 Collegium Medicum, Institute of Nursing, University of Rzeszów, Rzeszów, Poland; 2 Department of Integrated Medical Care, Medical University of Białystok, Białystok, Poland; 3 Department of Nursing, Institute of Health Protection, The Bronisław Markiewicz Academy of Applied Sciences, Jarosław, Poland

**Keywords:** assessment, depression, functional capacity, home care, MNA nutritional status

## Abstract

**Introduction:**

The growing number of chronically ill individuals and the ageing of the population increase the need for systematic assessment of nutritional status and functional capacity in patients receiving long-term care. Malnutrition, reduced independence, and depressive symptoms often co-occur and may constitute a significant challenge when planning care in the home setting.

**Aim:**

The aim of this study was to assess the co-occurrence of nutritional status, severity of depressive symptoms, and level of functional ability in chronically ill patients receiving long-term home care.

**Material and methods:**

A cross-sectional study was conducted among 234 patients receiving long-term home nursing care in Poland. A survey questionnaire and standardized instruments were used: the Barthel Index, the Mini Nutritional Assessment (MNA), and the Beck Depression Inventory (BDI). The analysis was performed using the Mann–Whitney U test and Spearman’s rho correlation (p ≤ 0.05). Due to the lack of clinical data on comorbidities and pharmacotherapy, no multivariate analyses were performed.

**Results and conclusions:**

In the present study, the mean Barthel Index score (~19.4 points) indicated significant limitations in performing basic activities of daily living. Among the respondents, 67.6% showed moderate to severe depressive symptoms, while 85.5% were classified as being at risk of malnutrition. Significant negative correlations were found between the BDI score and all components of the MNA (rho = –0.301 to –0.381; p < 0.001), indicating that poorer nutritional status co-occurred with greater severity of depressive symptoms. In the studied group of patients receiving long-term home care, the concurrent presence of functional limitations, risk of malnutrition, and depressive symptoms was frequently observed. Given the cross-sectional design of the study and the absence of adjusted analyses, the findings should be interpreted with caution and treated as a description of co-occurrence, without the ability to infer the direction of the relationships.

## 1 Introduction

The processes of aging, including age-related loss of muscle mass (sarcopenia), chronic non-communicable diseases (NCDs), multimorbidity, and inequalities in access to healthcare systems, predispose older individuals to malnutrition and loss of functionality. In the geriatric population, inadequate intake of energy and protein, reduced absorption of nutrients, and metabolic disturbances are frequently observed, which may lead to impaired immunity, delayed wound healing, cognitive decline, and an increased risk of complications. According to various diagnostic criteria, the prevalence of malnutrition among residents of nursing homes ranges from 20% to as high as 60%. Loss of self-care ability increases the demand for long-term care (LTC) services, particularly in middle- and high-income countries. However, the quality of these services varies widely both between and within countries. One of the key indicators of quality in institutional care is access to personalized nutritional care, tailored to the individual needs and risk profile of patients (1).

Personalized nutritional care plays an invaluable role in maintaining the health of older adults in nursing homes and long-term care facilities. However, the extent of implementation of a standardized process—from screening, nutritional assessment, and interventions to ongoing monitoring—varies greatly across the European Union, not only between countries and regions but also among local healthcare providers. Although clinical guidelines and evidence-based quality standards exist, comprehensive interdisciplinary approaches to personalized nutritional care for LTC residents are still implemented only to a limited extent in practice (2–4).

Nutritional status plays a critical role in the course and treatment of chronic diseases. Malnutrition, often underestimated in clinical practice, may result in health deterioration, increased incidence of complications, and prolonged hospital stays. It is estimated that between 20% and 60% of chronically ill patients are affected by malnutrition, depending on the underlying condition, age, and treatment approach (5–7).

Functional capacity – understood as the ability to perform basic activities of daily living – declines with the progression of chronic diseases and with age, increasing dependence on caregivers (8). Individuals with limited independence are more prone to deterioration in nutritional status due to difficulties in preparing and consuming meals, reduced physical activity, and muscle weakness (9).

Depressive symptoms represent another important component of the clinical profile of patients receiving long-term care. Depression affects as many as 40–60% of long-term care patients (10, 11) and may co-occur with loss of appetite, reduced activity, sleep disturbances, and decreased interest in food, which may be associated with lower energy and protein intake (12). At the same time, depressive symptoms have been reported to coincide with declines in physical functioning and phenomena corresponding to the frailty syndrome, whereas malnutrition and loss of independence may increase patients’ psychological burden—supporting the concept that these relationships are bidirectional (13, 14).

Numerous studies have demonstrated significant associations between worsening nutritional status, low functional capacity, and increased severity of depressive symptoms in older and chronically ill individuals (9, 11, 13). Comprehensive assessment of patients in long-term care requires consideration of all three components. Tools such as the Mini Nutritional Assessment (MNA), the Barthel Index, and the Beck Depression Inventory (BDI) allow standardized assessment of nutritional status, functional capacity, and severity of depressive symptoms, respectively (1, 5, 14).

In view of the mutual interactions between malnutrition, depression, and functional impairment, it is particularly important to deepen knowledge of the relationships among these areas in patients receiving long-term home care. This may provide a basis for designing interventional studies (15). In the present study, standardized instruments (the MNA, BDI, and the Barthel Index) were used to describe the co-occurrence of nutritional status, depressive symptoms, and functional ability in a population of patients receiving long-term home care.

The aim of the study was to assess the co-occurrence of nutritional status, severity of depressive symptoms, and level of functional ability in chronically ill patients receiving long-term home nursing care.

Based on the main aim, the following research questions were formulated:

1. Does the severity of depressive symptoms (BDI) co-occur with a lower level of functional ability (Barthel Index)?
2. Does the severity of depressive symptoms (BDI) co-occur with poorer nutritional status (MNA)?

## 2 Materials and methods

### 2.1 Research design

This study was conducted among patients receiving long-term home nursing care in south-eastern Poland. The diagnostic survey method was applied, using a questionnaire that included sociodemographic data as well as standardized research tools: the Barthel Index, the Mini Nutritional Assessment (MNA) for nutritional status assessment, and the Beck Depression Inventory (BDI). The study was conducted in the second half of 2024 and was observational and cross-sectional in nature.

### 2.2 Research tools

#### 2.2.1 Barthel Index

The Barthel Index is used to assess the patient’s level of independence in performing basic activities of daily living. Based on the score (ranging from 0 to 100), the level of functioning and the need for care can be determined. The diagnostic value and applicability of the Barthel Index in the Polish healthcare system have been confirmed in scientific studies.

The tool demonstrates high reliability and reproducibility of results, as evidenced by a Cronbach’s alpha coefficient ranging from 0.78 to 0.89, and a test–retest correlation coefficient in the range of R = 0.93–0.95. In clinical and institutional practice (e.g., in the qualification process for long-term care), particular significance is attributed to scores within the 0–40 range, which indicate severe functional deficits and justify the need for continuous care (8, 16).

#### 2.2.2 Mini nutritional assessment

The MNA is a validated and widely used tool for nutritional status assessment. It was developed to identify patients who are malnourished or at risk of malnutrition. The MNA enables early detection of nutritional problems, allowing for the implementation of appropriate interventions to improve patients’ quality of life. It includes questions concerning food intake, weight loss, mobility, psychological stress, and body mass index (BMI) (17). The tool consists of simple measurements and short questions, typically taking about 10 minutes to administer. The MNA assesses four key domains:

- Anthropometric measurements (weight, height, calf circumference, arm circumference),
- Food and fluid intake (appetite, intake of protein products, fruits and vegetables, number and type of meals, fluid intake),
- General assessment (living situation, mobility, stress or illness, medications, skin condition, neuropsychological issues),
- Subjective assessment (self-perception of health and nutritional status).
- The total MNA score distinguishes three groups:
- Normal nutritional status (24–30 points),
- At risk of protein–energy malnutrition (17–23.5 points),
- Malnourished (<17 points) (18).

#### 2.2.3 Beck depression inventory

The Beck Depression Inventory (BDI) is one of the most widely used self-assessment tools for depression worldwide. It was developed by Aaron T. Beck and colleagues in 1961 and has since undergone multiple modifications and cultural adaptations, including in Poland. The Cronbach’s α coefficient of the Polish adaptation is 0.92 (19, 20). The BDI is based on the cognitive theory of depression, which emphasizes negative beliefs about the self, the surrounding world, and the future. It is a self-report instrument, with patients independently assessing the severity of their symptoms by selecting responses that best describe their psychological state during the past seven days. The scale covers three main domains: emotional, cognitive, and somatic. It should be taken into account that some items of the BDI refer to somatic symptoms, which may co-occur with chronic diseases and could potentially affect the scale score.

### 2.3 Participants

The study included 234 patients receiving home care. The inclusion criteria were as follows: provision of informed consent to participate, the ability to answer the questionnaire items in a manner permitting analysis, and receipt of services within the framework of home-based long-term nursing care. The exclusion criteria included lack of consent to participate and return of an incomplete questionnaire that precluded analysis (e.g., missing key responses). Paper-based questionnaires were distributed by the authors, after obtaining approval from the managers of home care facilities in south-eastern Poland. During meetings with nursing staff, the purpose of the study and instructions for completing the questionnaire were provided, with a request to administer the survey in patients’ home settings. As part of the study, 300 questionnaires were distributed; 66 were either not returned or were returned in a form that precluded analysis (e.g., with missing key responses). Questionnaires were provided to patients by nursing staff delivering home-based long-term nursing care services. The Beck Depression Inventory (BDI) is a self-report measure and was completed independently by patients; when limitations were present (e.g., visual or manual difficulties or general weakness), support from an informal caregiver was permitted, while maintaining the confidentiality of responses. No standardized screening instruments were used to assess cognitive function (e.g., the MMSE or MoCA); therefore, it was not possible to determine the proportion of participants with cognitive impairment.

### 2.4 Ethical procedure

The study was conducted in accordance with ethical standards outlined in the Declaration of Helsinki (64th WMA General Assembly, Fortaleza, Brazil, October 2013) and Polish legal regulations. Approval was obtained from the Bioethics Committee of PANS in Przemyśl (approval number: KBPANS/17/2024).

### 2.5 Statistical analysis

Descriptive statistics were used in the analysis. Differences between groups were compared using the nonparametric Mann–Whitney U test. Associations between quantitative and ordinal variables were assessed using Spearman’s rho correlation coefficient. Statistical significance was set at p ≤ 0.05. Due to the lack of standardized clinical data on comorbidities, pharmacotherapy, and participants’ cognitive status, it was not possible to perform multivariable analyses (e.g., regression) to control for potential confounders. Consequently, the results should be interpreted as a descriptive analysis of the co-occurrence of the phenomena examined in the study population. All analyses were performed using IBM SPSS Statistics 26.0 with the Exact Tests module.

## 3 Results

The study included 234 patients receiving home care. The majority were women – 164 individuals (70.1%), while men accounted for 29.9% (n = 70). In terms of age, the dominant group were patients over 65 years of age – 158 individuals (67.5%). The remaining age groups were as follows: 46–55 years – 36 individuals (15.4%), 56–65 years – 26 individuals (11.1%), 26–35 years – 10 individuals (4.3%), and 36–45 years – 4 individuals (1.7%). A total of 122 patients (52.1%) lived in urban areas, whereas 112 (47.9%) lived in rural settings.

The duration of home care varied: most patients (62.4%, n = 146) had been under care for 1 to 5 years. Fifty patients (21.4%) had been receiving care for 6 to 10 years, 34 patients (14.5%) for less than one year, and only 4 patients (1.7%) for more than 10 years.

Regarding additional support provided apart from the long-term care nurse, in the vast majority of cases care was provided by immediate family members (88.0%, n = 206). Eighteen patients (7.7%) were supported by social welfare caregivers (MOPS/GOPS), six patients (2.6%) by extended family, while four patients (1.7%) remained without support, living alone.

Respondents reported a variety of diagnosed medical conditions that formed the basis for their inclusion in long-term home nursing care due to functional deficits. Among younger patients, the most common diagnoses were injuries resulting from traffic accidents, genetic disorders, neuromuscular diseases, and cancers. Among older patients, the main reasons for qualification were complications arising from the course of chronic diseases. It is worth noting that many patients also suffered from comorbidities, which further contributed to deterioration of their general health and consequently their nutritional status.

### 3.1 Results of the Barthel Index

Among the 234 participants, the majority were completely dependent (0–20 points on the independence assessment scale) – 150 individuals, accounting for 64.1% of the group. Partially independent individuals (21–40 points) represented 35.9%, corresponding to 84 patients. These data describe the level of functional dependence in the study group (**Table 1**). The mean Barthel Index score for the entire sample was 19.38 points, and the median was 15.00 (**Table 2**). No significant differences were found between women and men in the level of independence (U = 1360.0; p = 0.655; exact test).

**Table 1:** Scores obtained during the most recent assessment according to the Barthel index.

Level of independence		Frequency (N)	Percentage (%)
Valid	Completely dependent (0–20 pts)	150	64.1
	Partially independent (21–40 pts)	84	35.9
	Total	234	100.0

**Table 2:** Results of the Mann–Whitney U nonparametric statistical test by gender of respondents.

Gender		Score obtained during the most recent assessment according to the Barthel Index	Beck depression inventory (total score)	MNA – Overall nutritional status (max. 30)	MNA – Screening section (max. 14)	MNA – Patient assessment section (max. 16)
Female	Average	19.28	17.73	18.75	8.63	10.10
	Median	15.00	18.00	18.00	9.00	10.00
	Average rank	58.09	58.84	56.05	58.74	53.57
	Frequency (N)	164	164	164	164	164
	Standard deviation	14.05	9.98	4.45	2.73	2.48
	Minimum	0.00	0.00	5.50	2.00	3.50
	Maximum	65.00	45.00	27.50	14.00	16.00
Male	Average	19.60	17.49	19.87	8.71	11.16
	Median	20.00	19.00	20.50	9.00	12.00
	Average rank	61.14	59.39	65.90	59.61	71.71
	Frequency (N)	70	70	70	70	70
	Standard deviation	12.36	9.28	4.00	2.89	2.11
	Minimum	0.00	0.00	10.00	3.00	5.00
	Maximum	65.00	33.00	25.00	13.00	15.00
Total	Average	19.38	17.66	19.09	8.66	10.41
	Median	15.00	18.00	18.50	9.00	10.50
	Average rank	234	234	234	234	234
	Frequency (N)	13.51	9.74	4.33	2.76	2.42
	Standard deviation	0.00	0.00	5.50	2.00	3.50
	Minimum	65.00	45.00	27.50	14.00	16.00
Mann-Whitney U		Maximum	1421.500	1193.500	1413.500	990.000
p		0.653	0.936	0.150	0.898	0.008
p (exact)		0.655	0.937	0.151	0.899	0.007

### 3.2 Results of the mini nutritional assessment

In the studied group, the mean body weight was 75.22 kg (median: 75.00 kg), with a standard deviation of 13.70 kg. The lowest recorded body weight was 48.00 kg, while the highest was 120.00 kg. The mean height was 164.97 cm, with a median of 163.50 cm and a standard deviation of 7.90 cm (range: 150.00–187.00 cm). The mean body mass index (BMI) for the entire group was 27.72, suggesting overweight in the study population according to WHO classification. The median BMI was 26.92, with a standard deviation of 4.70. The lowest BMI value was 19.98 (normal), while the highest was 42.46 (class III obesity).

*MNA – Overall nutritional status (max. 30 points)*

Overall assessment of nutritional status classified 26.5% of patients (n = 62) as malnourished (≤16 points). A total of 59.0% (n = 138) were identified as being at risk of malnutrition (17–23.5 points), while only 14.5% (n = 34) achieved scores indicating normal nutritional status (24–30 points). The mean overall MNA score was 19.09, with a median of 18.50 and a standard deviation of 4.33. Scores ranged from 5.50 to 27.50. No significant differences were found between women and men in total MNA scale scores (U = 1193.5; p = 0.151).

*MNA – Screening section (max. 14 points)*

In the screening section of the MNA, 31.6% of patients (n = 74) scored between 0–7 points, indicating malnutrition. More than half of the respondents (51.3%, n = 120) scored 8–11 points, suggesting risk of malnutrition. Only 17.1% (n = 40) achieved scores between 12–14 points, corresponding to normal nutritional status. As in the overall assessment, the majority of patients (82.9%) required nutritional support, either preventive or interventional. The mean screening score was 8.66 (median: 9.00; SD = 2.76), with values ranging from 2.00 to 14.00. No significant differences were observed between women and men in MNA screening section scores (U = 1413.5; p = 0.899).

*MNA – Patient assessment section (max. 16 points)*

The mean patient assessment score was 10.41 (median: 10.50; SD = 2.42), with results ranging from 3.50 to 16.00. These findings further confirm variation in nutritional status among the studied patients. For the patient assessment score (maximum 16 points), a significant difference was observed between sexes—men achieved higher scores than women (U = 990.0; p = 0.007; exact test). Detailed mean values of the MNA questionnaire are presented in **Table 3**.

**Table 3:** Mean results of the MNA questionnaire (N = 234).

Variables	Mean	Median	Standard deviation	Minimum	Maximum
MNA – Overall nutritional status (max. 30)	19.09	18.50	4.33	5.50	27.50
MNA – Screening section (max. 14)	8.66	9.00	2.76	2.00	14.00
MNA – Patient assessment section (max. 16)	10.41	10.50	2.42	3.50	16.00

### 3.3 Results of the Beck Depression Inventory

The study included 234 patients whose results were classified according to the severity of depressive symptoms. Moderate or severe depressive symptom severity (according to the adopted classification) was identified in 67.8% of participants, indicating a substantial health problem in this patient group that warrants psychological or psychiatric support. Only 16.2% showed no signs of depression. No significant differences were found between women and men in the severity of depressive symptoms (U = 1421.5; p = 0.937; exact test).

### 3.4 Correlations

**Table 4** presents Spearman’s rho correlation coefficients between the total Beck Depression Inventory (BDI) score and three indicators of nutritional status:

**Table 4:** Associations between the beck depression inventory (BDI) score and MNA scale scores (Spearman’s rho correlation), *n* = 234.

Variable	Spearman’s rho	p (two-tailed)
MNA – total nutritional status score (max. 30 pts)	–0.381	<0.001
MNA – screening section score (max. 14 pts)	-0.301	<0.001
MNA – patient assessment score (max. 16 pts)	-0.322	<0.001

- MNA – Overall nutritional status (max. 30 points): The correlation coefficient was rho=–0.381 (p < 0.001), indicating a moderate, negative relationship – an inverse relationship was observed between the scores.
- MNA – Screening section (max. 14 points): The correlation was also significantly negative at rho=–0.301 (p < 0.001), showing that weaker screening scores were associated with higher depression levels.
- MNA – Patient assessment section (max. 16 points): The coefficient reached rho=–0.322 (p < 0.001), again confirming a significant relationship – poorer functional-nutritional status correlated with greater depressive symptomatology.

All three analyzed relationships showed statistically significant negative correlations. This means that lower MNA scores (poorer nutritional status) were accompanied by higher BDI scores (greater severity of depressive symptoms). These findings should be interpreted as indicating co-occurrence of the phenomena examined; due to the cross-sectional design of the study, it is not possible to determine the direction of the relationships or to draw causal inferences (**Table 4**).

**Table 5** presents the values of Spearman’s rho correlation coefficients for two independent variables—age and duration of care—in relation to five dependent variables.

**Table 5:** Correlations between the barthel index, beck depression inventory (BDI), MNA scores and age and duration of home care (Spearman’s rho correlation).

Variable	Age (rho)	p	Duration of care (rho)	p
Barthel Index	-0.073	-0.047	–0.047	0.484
Beck Depression Inventory (BDI)	-0.033	-0.002	–0.002	0.974
MNA – Overall nutritional status (max. 30)	0.072	0.026	0.026	0.698
MNA – Screening section (max. 14)	0.117	0.104	0.104	0.111
MNA – Patient assessment section (max. 16)	0.007	-0.060	–0.060	0.363

1. For the Barthel Index (patient functionality), the correlation with age was rho=–0.073 and with duration of care rho=–0.047. Both correlations were very weak and negative, indicating only a minimal tendency toward decreased independence with increasing age and longer duration of care.
2. For the Beck Depression Inventory (BDI), the correlation with age was rho=–0.033 and with duration of care rho=–0.002. These values are essentially zero, suggesting no meaningful association between either age or duration of care and depressive symptom severity.
3. For the MNA – Overall nutritional assessment (max. 30 points), correlations were rho=0.072 (age) and rho=0.026 (duration of care). Both values suggest very weak positive associations, indicating that slightly higher age and longer care duration may be marginally related to better nutritional assessment, though the effect is negligible.
4. For the MNA – Screening section (max. 14 points), correlations were the highest in the table: rho=0.117 (age) and rho=0.104 (duration of care). Although still weak, these values suggest a small trend that older patients and those under care for longer achieved somewhat better MNA screening results, which could reflect the effectiveness of long-term care in maintaining nutritional status.
5. For the MNA – Patient assessment section (max. 16 points), correlations were rho=0.007 (age) and rho=–0.060 (duration of care), confirming the absence of significant associations.

Overall, all analyzed correlations were very weak and statistically non-significant (p > 0.05). Neither age nor duration of care was significantly associated with the Barthel Index, the Beck Depression Inventory, or MNA outcomes.

Using the nonparametric Mann–Whitney U test, no statistically significant gender-related differences were observed in the level of independence assessed with the Barthel Index among patients receiving long-term care. Both women and men obtained comparable results, with small differences in medians and means that did not reach statistical significance (p = 0.653).

Similarly, the severity of depressive symptoms did not differ significantly between genders. Female and male respondents achieved nearly identical BDI scores, with differences that were negligible and statistically non-significant (p = 0.936).

With respect to nutritional status (both the full and screening MNA assessments), men achieved slightly higher scores, indicating a tendency toward better nutritional status. However, these differences were not statistically significant (full MNA: p = 0.150; screening section: p = 0.898).

The only statistically significant gender difference emerged in the MNA – Patient Assessment subscale (max. 16 points). Men obtained significantly higher scores than women (mean 11.16 vs. 10.10), p = 0.008. The potential reasons for this difference cannot be definitively assessed on the basis of the data collected in this study and require further investigation in future projects. Detailed results are presented in **Table 2**.

## 4 Discussion

The aim of this study was to assess the co-occurrence of nutritional status, severity of depressive symptoms, and functional ability in chronically ill patients receiving long-term home care. The results indicate that, in the study population, significant functional limitations, high severity of depressive symptoms, and risk of malnutrition frequently occurred concurrently. Given the cross-sectional design and the lack of adjusted analyses, the findings should be interpreted as a description of co-occurring relationships, without the possibility of determining the direction of associations between variables.

In our research, the mean score on the Barthel Index (~19.4 points) indicated significant limitations in performing basic activities of daily living. Comparable results in populations of long-term care patients have been reported, consistently demonstrating a high degree of dependency (21, 22). The Barthel Index is a well-validated instrument, with inter-rater reliability exceeding 0.90, confirming its applicability also in home care settings (23). No significant differences in functional status were observed between women and men (p = 0.653), which is consistent with prior observations that functional capacity is more strongly influenced by comorbidities and biological age than by gender (24). It is worth noting that functional performance may additionally be modulated by nutritional status and psychological well-being, as highlighted in previous studies (23).

In this study, 67.6% of patients presented with moderate to severe depressive symptoms. The Beck Depression Inventory (BDI) is a widely used tool, and its Polish adaptation demonstrates high reliability (Cronbach’s α = 0.92) (25). The absence of significant gender differences in depressive symptoms (p = 0.936) aligns with evidence suggesting that, in older populations, the influence of biological and social determinants outweighs gender-specific factors (26). Depression in elderly patients frequently coexists with chronic somatic diseases and reduced appetite, further affecting general health status (27). In the study population of patients receiving home-based long-term care, a high burden of chronic diseases should also be considered, including neurological, oncological, and dementing disorders, which may confound the observed associations. The lack of a detailed comorbidity analysis limits the ability to determine which conditions had the greatest impact on the occurrence of depression and the risk of malnutrition. It should be emphasized that, in a population of chronically ill patients, interpretation of BDI results requires caution, because some items on the scale refer to somatic symptoms (e.g., fatigue, sleep disturbances, loss of appetite) that may result both from low mood and from the course of physical illnesses. Therefore, the obtained results may partly reflect the overall burden of chronic disease rather than solely the severity of depression in the clinical sense.

Our findings revealed that 85.5% of patients were at risk of malnutrition. This result corresponds with previous studies conducted in home and institutional long-term care, where the prevalence of malnutrition risk reached up to 90% (28). Other studies have reported that while the prevalence of malnutrition was not consistently higher in older adults, approximately half were at risk, with variation depending on gender, study setting, and region. These findings underscore the need for further studies employing diverse nutritional assessment tools, as well as interventional research in older populations (29). In our study, no statistically significant gender differences were observed in overall MNA scores, suggesting that the risk of malnutrition is a general phenomenon across the elderly patient population, regardless of gender.

The analysis demonstrated statistically significant negative correlations between the BDI score and all components of the MNA. Results indicate associations of weak to moderate strength. This means that the co-occurrence of poorer nutritional status with greater severity of depressive symptoms is evident at the population level; however, it does not represent a strong or deterministic relationship. Moreover, given the cross-sectional design of the study and the inability to control for comorbidities and pharmacotherapy, it is not possible to determine the extent to which the observed associations are independent of potential confounding factors. Similar associations have been documented in large geriatric populations, where malnutrition was found to increase the risk of depression up to threefold (30).

It should be emphasized that, due to the cross-sectional design of the study, the findings are correlational and do not allow causal inference or determination of the direction of the association between the severity of depressive symptoms and nutritional status. Both directions are plausible: depression may contribute to poorer nutrition (e.g., through reduced appetite, diminished motivation, and lower activity levels), and conversely, deterioration in nutritional status may exacerbate depressive symptoms. The influence of third factors (e.g., comorbid conditions) that may simultaneously affect both the mental and physical health of patients also cannot be ruled out.

Moreover, meta-analyses emphasize that improvements in nutritional status can substantially reduce depressive symptoms in older adults (25). Findings from other studies suggest that nutritional interventions may influence psychological well-being; however, the present study does not allow the effects of such interventions to be assessed (31). In this study, the strong association between MNA and BDI results reinforces this need.

The only significant gender difference was observed in the “MNA – patient assessment score” subscale (p = 0.007), where men achieved higher values. This may reflect better functioning in terms of appetite, self-feeding, and overall physical well-being. Comparable results have been reported in other studies, where older women more frequently presented with limitations in food intake and reduced appetite (32).

Overall, the findings highlight a strong interrelationship between three key domains: functional status, nutritional condition, and depressive symptoms. In clinical practice, a comprehensive assessment of patients in long-term care may encompass nutritional status, functional capacity, and psychological functioning; however, evaluating the effectiveness of interventions requires prospective studies. This finding may indicate sex-related differences in subjective assessment of nutritional status; nonetheless, the interpretation should be made with caution, as potential explanatory variables were not measured in the study (33). These results are consistent with the current state of knowledge, according to which depression, malnutrition, and functional impairment mutually exacerbate one another, creating a vicious circle that leads to poorer prognosis and an increased need for care (13). In view of the above, it is justified to implement individualized care with particular emphasis on individuals with pronounced depressive symptoms and limited functional capacity. Such an approach may help reduce the risk of complications, and optimize the costs of long-term care.

In the context of organizing home-based long-term care in Poland, it should be noted that in many cases patients’ primary support is provided by informal caregivers, with limited availability of specialist services (e.g., a clinical dietitian or psychologist) in the home setting. This may affect both the prevalence of malnutrition risk and the frequency of unrecognized and untreated depressive symptoms. The results of the present study indicate a need to implement more integrated approaches to the assessment and monitoring of mental health and nutritional status within home-based long-term nursing care practice.

## 5 Conclusion

In the studied group of patients receiving long-term home care, a low level of functional ability and a high prevalence of risk of malnutrition and depressive symptoms were observed. Statistically significant negative correlations were found between MNA and BDI scores, indicating that poorer nutritional status co-occurred with greater severity of depressive symptoms. Due to the cross-sectional design and the lack of adjusted analyses, the results do not allow for causal inference or determination of the direction of the relationships between the variables studied.

## 6 Limitations of the study

The study has limitations typical of a cross-sectional design, which precludes causal inference and determination of the direction of relationships between the severity of depressive symptoms and nutritional status and functional ability. Standardized data on comorbidities and pharmacotherapy were not collected, which made it impossible to perform multivariable analyses and to control for potential confounding factors. No screening tools were used to assess cognitive function (e.g., MMSE/MoCA), and some questionnaires were completed with the assistance of a caregiver, which may have affected the reliability of responses in self-report scales. In addition, some items of the BDI refer to somatic symptoms which, in a chronically ill population, may partially overlap with symptoms of underlying conditions. The study included patients from a single region of Poland, which may limit the generalizability of the findings.

## 7 Implications for practice

The findings indicate that, in patients receiving long-term home care, systematic monitoring of nutritional status and screening for depressive symptoms are warranted, particularly in individuals with significant functional limitations. In the home-care setting, routine use of simple, standardized assessment tools (the Barthel Index, MNA, and BDI) may be useful for identifying patients who require more in-depth diagnostics and nutritional or psychological support. Evaluation of the effectiveness of specific interventions (nutritional and psychological) requires further prospective and interventional studies.

## Data Availability

The raw data supporting the conclusions of this article will be made available by the authors, without undue reservation.
